# Reactive Oxygen Species (ROS)-Responsive Biomaterials for the Treatment of Bone-Related Diseases

**DOI:** 10.3389/fbioe.2021.820468

**Published:** 2022-01-11

**Authors:** Xiaoxiang Ren, Han Liu, Xianmin Wu, Weizong Weng, Xiuhui Wang, Jiacan Su

**Affiliations:** ^1^ Institute of Translational Medicine, Shanghai University, Shanghai, China; ^2^ Department of Orthopedics, Zhongye Hospital of Shanghai, Shanghai, China; ^3^ Department of Orthopedics Trauma, Shanghai Changhai Hospital, Naval Military Medical University, Shanghai, China

**Keywords:** bone remodeling, ROS-responsive biomaterials, bone-related diseases, bone regeneration, photodynamic therapy

## Abstract

Reactive oxygen species (ROS) are the key signaling molecules in many physiological signs of progress and are associated with almost all diseases, such as atherosclerosis, aging, and cancer. Bone is a specific connective tissue consisting of cells, fibers, and mineralized extracellular components, and its quality changes with aging and disease. Growing evidence indicated that overproduced ROS accumulation may disrupt cellular homeostasis in the progress of bone modeling and remodeling, leading to bone metabolic disease. Thus, ROS-responsive biomaterials have attracted great interest from many researchers as promising strategies to realize drug release or targeted therapy for bone-related diseases. Herein, we endeavor to introduce the role of ROS in the bone microenvironment, summarize the mechanism and development of ROS-responsive biomaterials, and their completion and potential for future therapy of bone-related diseases.

## Introduction

As an internal support system, bone is a dynamic connective tissue with highly mineralized architecture giving it substantial strength, providing a structural foundation for the human body and muscle (McEnery et al., 2016). Bone is in the dynamic modeling and remodeling processes by developing the activities of bone formation and resorption, for the healthy development of the skeleton (Florencio-Silva et al., 2015). With the increased longevity of human life and the aging of population, bone-related diseases, such as osteoporosis, osteosarcoma, bone metastasis, osteoarthritis, and osteomyelitis, cause significant pain and amplify the economic burden for millions of people worldwide. Growing evidence indicates that bone homeostasis is adversely affected by oxidative stress induced by reactive oxygen species (ROS), thus targeting ROS might be a vital approach for the treatment of bone metabolic disorders (Agidigbi and Kim, 2019). In the meantime, ROS are necessary for bone remodeling machinery as they can enhance the degradation of the mineralized matrix and affect the behaviors of all the cells involved in bone remodeling. ROS is normally divided into two categories: non-radical derivatives of oxygen and free chemically reactive oxygen radicals (Phaniendra et al., 2015). ROS production could lead to an increased level of oxidative stress, which increases the risk of mutations in mitochondrial and nuclear DNA (Russo et al., 2012). Besides, oxidative stress has been known as a major donor to the immune response and linked with the pathophysiology of almost all organs (Mittal et al., 2014). It has also been proved that oxidative stress play role in aging and lead to degenerative diseases with increasing age (Sharifi-Rad et al., 2020). Therefore, it is important to understand how ROS modulate bone biology and pathology.

In addition, for the therapy of bone-related diseases, researchers are developing various scaffolds, hydrogels, nanocarriers, and drugs (Yao et al., 2019; Yin et al., 2021; Zheng et al., 2021). Synthetic or natural biomaterials can enhance the restoration of bone structure and function. Implant materials make contact with the patient’s tissue, permitting interactions with endogenous bone. Among all kinds of treatments, developing novel biomaterials and drugs which targeted the high-level ROS should be promising solutions for bone-related diseases. The antioxidants or selected therapeutic compounds can be loaded with biomaterials and will be stimuli-responsive released with the presence of oxidation. Hubbel’s team was the first to use ROS-responsive biomaterials for drug delivery in 2001 (Napoli et al., 2001), and then this new strategy quickly spreads for different biomedical applications. However, the summary of the recently developed strategies about ROS-responsive biomaterial for bone-related diseases is still lacking. In this review, we will introduce the major findings of the regulatory role of ROS in osteoclast biology, including the influence of ROS on signaling, proliferation, and differentiation of bone cells. The review will also mention the ROS-responsive biomaterials which could be excellent options sense and respond ROS microenvironment. How targeting ROS may be a solution for the therapy of bone-related diseases, such as osteoarthritis, will be discussed. Finally, we propose a prospect for the trends and future development of ROS-responsive biomaterials for the application in bone tissue.

## Reactive Oxygen Species in Bone Remodeling

Bone is a dynamic organ to achieve the functions such as growth, movement, organ protection, or calcium/phosphate equilibrium (Hardy and Fernandez-Patron, 2020). Bone remodeling is mediated by the progress of formation and resorption led by the activities of osteoblast and osteoclast, allowing bone growth and tissue regeneration (Callaway and Jiang, 2015). In bone tissues, ROS generation is an essential signal in living organisms which could regulate cell functions, mediate inflammation, and affect the pathophysiology of tissues (Wauquier et al., 2009). Among all forms of ROS, the majority of forms related to the osteoclastogenesis are superoxide (O_2_^−^) and hydrogen peroxide (H_2_O_2_). Early research in the 1990s was about the first connecting ROS and osteoclast function by assessing the osteoclast activity after adding oxidants or antioxidant enzymes, indicating that the superoxide radical could enhance bone resorption (Garrett et al., 1990). ROS originated from nicotinamide adenine dinucleotide phosphate (NADPH) oxidase (Nox) were localized to the interface of bone-osteoclast, resulting in the production of superoxide within the osteoclast (Key et al., 1990). At the same time, other studies indicated that H_2_O_2_ was the main ROS to promote the formation and activity of osteoclast (Kim et al., 2006). However, different from these studies describing a direct role of ROS in bone resorption, subsequent studies suggested that ROS could promote osteoclast formation and activity *via* an indirect mechanism in activating signaling pathways (Hall et al., 1995). Specifically, ROS could influence several signaling pathways such as nuclear factor κB (NF-κB) and produce the following receptor activator of NF-κB ligand (RANKL).

**FIGURE 1:**
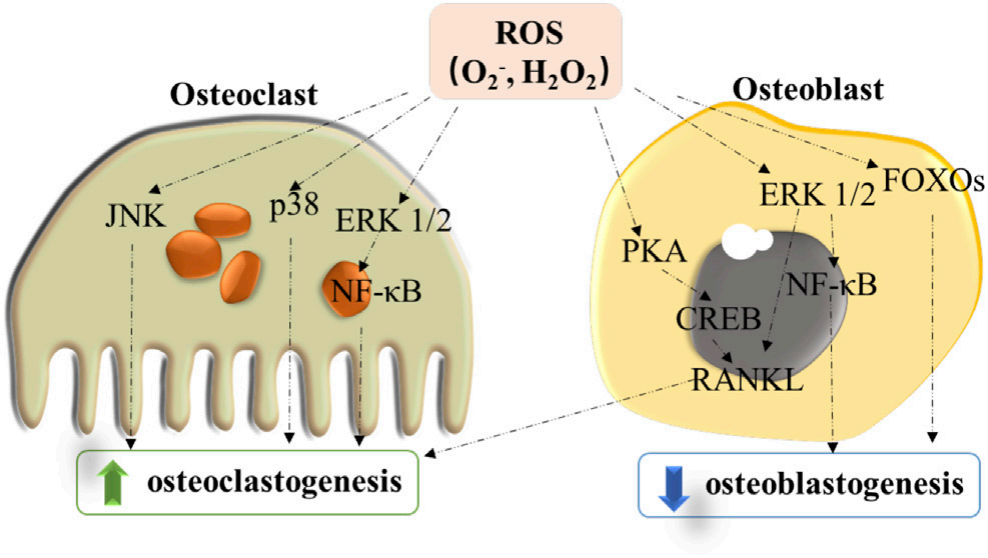
Modulation role of ROS for signaling pathways in inhibiting osteoblast differentiation and enhancing osteoclastogenesis.

Upon to the enhanced activity of osteoclast by ROS, H_2_O_2_ has shown an inhibition effect on the cell differentiation of osteoblast activities. Oxidative stress originated from H_2_O_2_ restrained the process of osteoblastic differentiation in the bone marrow stromal cells (BMSCs) of mouse and rabbit (Bai et al., 2004), showing a reduction in the alkaline phosphatase (ALP) activity. In addition to influencing the differentiation process, ROS also affects the lifespan of osteoblast (Linares et al., 2009). Thus, as shown in Figure 1, ROS not only directly promotes osteoclastogenesis but also inhibits the differentiation and growth of osteoblasts by stimulating RANKL-induced formation of osteoclast. ROS seems to perform an important role in bone by affecting both cell types. These findings also indicate that the treatment strategies for bone diseases by targeting ROS should be promising.

## The Mechanism of Reactive Oxygen Species-Responsive Biomaterials

**FIGURE 2:**
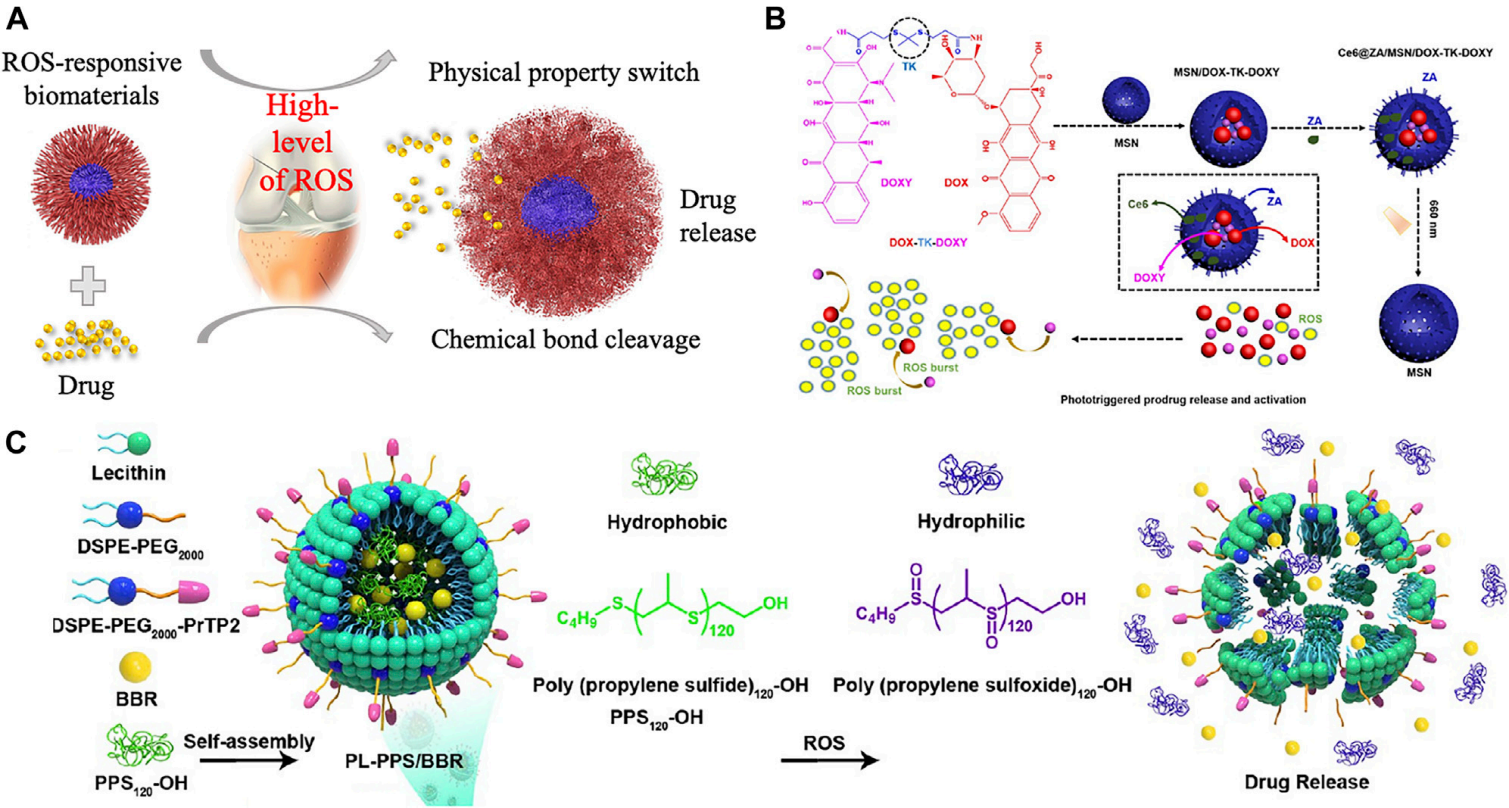
**(A)**. Scheme of ROS-responsive drug-loaded biomaterials and its responsive mechanism for the treatment of bone-related diseases. **(B)**. Prodrug DOX-TK-DOXY was loaded into the mesoporous silica nanoparticles (MSNs) and disrupt the TK linkage of the prodrug in an ROS environment. Reproduced with permission (Tong et al., 2020). Copyright 2020 American Chemical Society. **(C)** PPS_120_ converted to poly(propylene sulfoxide)_120_ and released drug in an ROS environment. Reproduced with permission (Zhao et al., 2021). Copyright 2021 American Chemical Society.

ROS are overproduced in diseased cells, and this property has been employed to develop ROS-responsive biomaterials as an intelligent drug delivery system. In response to ROS, ROS-responsive biomaterials can release drugs, used as targeting delivery agents, imaging agents, and therapeutic agents for regulating the tissue microenvironments and tissue regeneration. As described in Figure 2, mechanisms of the responsive function for ROS-responsive biomaterials can be classified into two types: ROS affect the physical properties of biomaterials especially affect their solubility, or ROS mediate the chemical properties which will lead to the bond cleavage reaction.

ROS can affect the physical property, especially the solubility of poly(propylene sulfide) (PPS), thioether-containing polymers, tellurium-, and selenium-containing polymers, favoring the drug release property (Gao and Xiong, 2021). In the presence of an oxidative environment, the hydrophobic sulfides can convert into hydrophilic sulfoxides and sulfones, contributing to their increased water solubility and potential application for drug delivery (Napoli et al., 2004). Poly(ethylene glycol) (PEG)-b-PPS and PPS-b-pyromellitic dianhydride (PMDA) copolymers are sensitized to H_2_O_2_, but shows no response against superoxide. While the PPS–PEG–superoxide dismutase (SOD) micelles are capable to turn into hydrophilic under both superoxide and hydrogen peroxide. In addition, thioether-based biomaterials also will be oxidized under the ROS environment. When susceptible to ROS (100 mM H_2_O_2_), the hydrophobic backbone of thioether containing biomaterials will be transformed to hydrophilic (Mahmoud et al., 2011). When simulated the ROS environment with 1 mM H_2_O_2_, a fast release of berberine (BBR) was observed from PL-PPS/BBR at 48 h (Figure 2C). During this progress, PPS_120_ scavenged ROS and converted to poly(propylene sulfoxide)_120_ when reacted with ROS to obtain the rapid release of BBR (Zhao et al., 2021). Thioether-containing polymers contribute an effective method for loading and delivering hydrophobic drugs especially for the application of cancer therapy. Selenium-containing copolymers show a similar solubility change under ROS as PPS, but more sensitive to ROS due to the bigger lower bond energy and larger radius of the selenium atom, making selenium-containing polymers a promising material for drug delivery. It was reported that the diselenide-containing micelles could react with ROS at a low concentration of H_2_O_2_ (0.01% v/v) and release drug in a short time (Xu et al., 2013).

In addition to the oxidant-induced solubility conversion, ROS can cleave the chemical bonds linkage of poly(thioketal) (TK), poly(proline), phenylboronic acid, and ester-containing polymers, giving rise to their degradation under the ROS environment. TK-containing polymers can be prepared by directly condensing polymerization using thiols for cancer therapy. With the degradation of thioketal linkages after exposure to 0.2 mM KO_2_, poly(1,4-phenylene-acetone dimethylenethioketal) (PPADT) has been used for the oral delivery of TNF-α-siRNA to the intestine of mice to treat ulcerative colitis (Wilson et al., 2010). Another ROS and pH dual-responsive PPADT-based biomaterial was developed for antiinflammatory therapy, showing 50% drug releasing in response to 1 mM H_2_O_2_ within 4 h (Pu et al., 2014). The poly(thioketal urethane) (PTK-URs) scaffolds are degraded in contact with the ROS released by cells, showing promising applications for wound repair (Martin et al., 2014) and bone regeneration (McEnery et al., 2016).

Among the various functional groups, phenyboronic acid and ester are unique because they are highly selectively and rapidly oxidized in responsive to H_2_O_2_ and generate phenol and boronic acid (Li et al., 2017). Being a polymer backbone or connected through an ether linkage were two strategies to include the groups in ROS sensitivity polymers which can be degraded in H_2_O_2_ (50–100 mM). An ROS-responsive nanocarrier (3I-NM@siRNA), which achieve the ROS-responsive property by adding the arylboronic ester group, was designed to carry small interfering RNA (siRNA) to improve long circulation stability of siRNA and increase their delivery efficiency (Zheng et al., 2019). Proline, as one of the amino acids, could also be cleaved by oxidation because of the metal-catalyzed oxidation of the proline segments, which make them a good candidate for ROS-responsive biomaterials (Amici et al., 1989).

## Reactive Oxygen Species-Responsive Biomaterials for Treating Bone-Related Diseases

Osteoarthritis (OA) is the most common form of arthritis, occurring most frequently in the population aged over 60 years (Shane Anderson and Loeser, 2010). OA was a degenerative joint disease and referred as an imbalance between the decay and formation of chondrocytes, extracellular matrix (ECM), and subchondral bone with a main feature of the destruction of cartilage (Sharma et al., 2013). Many factors could increase the risk of OA, including biomechanical forces, ROS, and auto-immunity (Corti, 2003). ROS is suspected in the pathological variation of microenvironments. The excessive ROS in OA will induce the damage of DNA and chondrocytes, and affect the production and turnover of ECM by stimulating matrix metalloproteinases (MMPs). In addition, the excess ECM production will stimulate immune cells to generate more ROS (Khan et al., 2017).

Since there is a positive relation between the high-level ROS and OA, many antioxidants which could scavenge the ROS are used to suppress inflammatory response in OA, including vitamin C, polysaccharides, or polymers without drugs. Polyphenols are derived from plants such as tea or grape, and have high antioxidant property, which have been extensively applied in bone regeneration and capsulated in a layer-by-layer coated gelatin nanoparticle (Shutava et al., 2009). To obtain a longer site-specific retention time, the ROS scavenger such as nitroxide radical compounds were loaded into triblock copolymers. Sponge polymeric microsphere (PPS-MS) was prepared to treat post-traumatic osteoarthritis (PTOA) by inhibiting the MMP activity and the destruction of articular cartilage (O’Grady et al., 2018).

Traditional therapy for OA requires frequent drug administration due to the quick removal speed. The drug delivery systems maintain the concentration of drugs and achieve specific release at the desirable site. The high concentration of ROS in OA can play as a trigger for the drug release from ROS-responsive biomaterials. For example, hollow poly(lactide-co-glycolide) (PLGA) microspheres were designed to treat OA for the delivery of antiinflammatory drug dexamethasone, FeCl_2_, sodium bicarbonate, and ethanol. PLGA shells allow the penetration of H_2_O_2_ and excessive H_2_O_2_ will active Fenton’s reaction, leading ethanol transform into acetic acid, thus making the sodium bicarbonate generate CO_2_ gas to breaks the PLGA shells and release dexamethasone. Compared to free dexamethasone, the responsive drug-loaded biomaterials show significant therapeutic effects (Chung et al., 2015).

Though the presence of ROS would worsen the condition in OA, ROS is required during the early phase of the antimicrobial (Ren et al., 2020a; Ji et al., 2021; Wang et al., 2021; Zhu et al., 2021) and anticancer (Ren et al., 2020b) progress for osteomyelitis and osteosarcomas. Particular emphasis is placed on the ROS-mediated photochemical and molecular mechanisms that give rise to the establishment of photothermal therapy as a treatment of highly resistant diseases, especially invasive and metastatic tumors. The inadequate ROS suppression could hinder the healing but the redox balance needs to be carefully maintained during the tissue regeneration phase.

The photodynamic therapy is a recently developed treatment that produces ROS from photosensitizers or photosensitizing agents by light activation to kill cancer cells or bacteria (Shi et al., 2019). Hu et al. (2019) reported a lipid-polymer hybrid nanocarrier with an ROS-responsive core consisted of homopolymer poly(thioketal phosphoester) (TK-PPE) to encapsulate chlorin e6 (Ce6) and DOX for the treatment of cancer. With the irradiation of 660 nm laser, the encapsulated Ce6 will generate ROS, inducing PDT for cancer treatment and rapidly degrades TK-PPE to burst release the loaded DOX, leading to an efficient combinational therapy of chemo-PDT to inhibit the growth of tumor *in vitro* and *in vivo* (Hu et al., 2019). Similarly, a size changeable HBPTK-Ce6 was designed by conjugating Ce6 onto the micelles containing thioketal units and loaded the anticancer drug camptothecin (CPT). The irradiation of 660 nm laser lead to the size reducing of HPBTK-Ce6@CPT and boost CPT release, which contribute a much deeper penetration in tumor to kill inner tumor cells compared with the original large-size micelles (Jin et al., 2019). More recently, bone-targeting prodrug mesoporous silica-based nanoreactor was developed to treat osteosarcoma. Upon laser irradiation, the loaded Ce6 produces *in situ* ROS and disrupt the TK linkage to release DOX and doxycycline (DOXY) from the prodrugs (Figure 2B) (Tong et al., 2020).

## Prospect and Conclusion

Many bone-related diseases such as osteoporosis, rheumatoid arthritis, and bone metastases have been linked to the states of higher ROS, thus understanding how ROS interacts with osteoclasts and other cells in the bone could enhance the development of novel therapeutic biomaterials to target ROS in the bone tissue. Antioxidant compounds may be beneficial to bone health, such as dried plum polyphenols (Bu et al., 2008) or simvastatin (Moon et al., 2011). Although it seems promising to use antioxidant agents to target ROS and treat bone diseases, ROS is present over the whole body. Therefore, the non-specificity ROS targeted compounds may affect other tissues, which are not limited to bone. To specifically treat the bone-related disease, biomaterials which could specifically target bone with ROS responsible property should be focused in the future. The combination with bone targeted drug such as bisphosphonates, oligopeptides, or tetracycline could be a prospective solution. In addition, adapter with ligand-receptor binding with the bone site could be decorated with the surface of ROS-responsive biomaterials. Moreover, multiple responsive carriers can be developed by the combination of ROS-responsive system with functional groups to achieve more accurate controlled release of drugs.

To sum up, ROS-responsive biomaterials have advantages when mediating oxidative stress-related diseases as drug delivery carriers or therapeutic agents, and thus should be a promising strategy for regulating bone microenvironments and bone regeneration.
